# Impact of renal function on the underlying pathophysiology of coronary plaque composition in patients with type 2 diabetes mellitus

**DOI:** 10.1186/s12933-017-0618-3

**Published:** 2017-10-12

**Authors:** Kentaro Kakuta, Kaoru Dohi, Miho Miyoshi, Takashi Yamanaka, Masaki Kawamura, Jun Masuda, Tairo Kurita, Toru Ogura, Norikazu Yamada, Yasuhiro Sumida, Masaaki Ito

**Affiliations:** 1 Department of Cardiology and Nephrology, Mie University Graduate School of Medicine, 2-174 Edobashi, Tsu, 514-8507 Japan; 2 Department of Cardiology, JCHO Yokkaichi Hazu Medical Center, Yokkaichi, Japan; 3 Clinical Research Support Center, Mie University Hospital, Tsu, Japan; 4 Department of Diabetes and Endocrinology, JCHO Yokkaichi Hazu Medical Center, Yokkaichi, Japan

**Keywords:** Mean amplitude of glycemic excursion, Serum tumor necrosis factor-α, Coronary plaque composition, iMap-IVUS, Renal function

## Abstract

**Background:**

Both the progression of diabetic kidney disease and increased glycemic variability play important roles in the pathogenesis of coronary plaque formation via inflammatory pathways in patients with type 2 diabetes mellitus (T2DM). Therefore we evaluated the role of renal function in the contributory effects of blood glucose fluctuations and blood levels of inflammatory cytokine concentrations on the tissue characteristics of coronary plaques in patients with T2DM.

**Methods:**

We prospectively enrolled 71 T2DM patients (mean age: 68 ± 9, male 79%) with 153 coronary artery lesions. Patients were divided into 2 groups according to their estimated glomerular filtration rate (eGFR) levels: Group 1 (≥ 60 mL/min/1.73 m^2^, n = 40) and Group 2 (< 60 mL/min/1.73 m^2^, n = 31). All patients underwent continuous glucose monitoring (CGM) for 120 h and the mean amplitude of glycemic excursions (MAGE) was calculated. Serum tumor necrosis factor (TNF)-α was also measured. In addition, gray-scale coronary intravascular ultrasound (IVUS) and iMap-IVUS were performed in the coronary lesions with < 50% luminal reduction.

**Results:**

In Group 1, MAGE correlated with percent lipidic volume (%LV) (r = 0.477, p = 0.002). In this group, stepwise multivariate linear regression analyses showed that only MAGE was independently associated with %LV (β = 0.477, p = 0.002). In contrast, in Group 2, only serum TNF-α correlated with percent fibrotic volume (%FV) (r = − 0.471, p = 0.007), %LV (r = 0.496, p = 0.005) and percent necrotic volume (%NV) (r = 0.426, p = 0.017). In this group, stepwise multivariate linear regression analyses showed that only serum TNF-α was independently associated with each tissue characteristic (%FV β = − 0.471 and p = 0.007, %LV β = 0.496 and p = 0.005, %NV: β = 0.426 and p = 0.017).

**Conclusions:**

In T2DM patients, the tissue characteristics of coronary plaques were associated with MAGE in patients with eGFR ≥ 60 mL/min/1.73 m^2^ and with serum TNF-α in those with eGFR < 60 mL/min/1.73 m^2^.

**Electronic supplementary material:**

The online version of this article (doi:10.1186/s12933-017-0618-3) contains supplementary material, which is available to authorized users.

## Introduction

Glycemic variability plays an important role in coronary plaque formation mainly via inflammatory pathways in patients with type 2 diabetes mellitus (T2DM). Recent clinical studies have shown that high glucose fluctuation strongly contributes to increased coronary plaque vulnerability both at the culprit lesion of acute coronary syndrome (ACS) [1, 2] and at unrelated lesions [3–5]. The pathogenesis of diabetic kidney disease is also closely linked to chronic inflammatory processes and may contribute to subsequent progression of coronary atheromatous plaques. Therefore, the contribution of glycemic variability to the tissue characteristics of coronary plaques may differ depending on the renal function in T2DM. Gray-scale intravascular ultrasound (IVUS) is a popular intracoronary imaging modality for the evaluation of coronary plaques in the clinical setting. iMap-IVUS is the most recent radiofrequency-IVUS system to incorporate a 40-MHz rotating single element catheter [6]. It can classify coronary plaques into fibrotic, lipidic, necrotic or calcified components using spectral radiofrequency analysis with a classification algorithm generated from ex vivo histological findings [6, 7]. Accordingly, the aim of the present study was to determine whether the factors that contribute to the tissue characteristics of coronary plaques differ depending on renal function in patients with T2DM using iMap-IVUS.

## Methods

### Patient selection

The study group consisted of 77 patients with T2DM over 20 years old who underwent invasive coronary angiography (ICA) to evaluate coronary artery disease (CAD) in Yokkaichi Hazu Medical Center between March 2015 and October 2016. Among them, 1 patient with end-stage renal disease with an estimated glomerular filtration rate (eGFR) < 15 ml/min/1.73 m^2^, 2 patients with ACS and 1 patient with prior coronary artery bypass grafting were excluded from the study. Therefore, 73 patients underwent continuous glucose monitoring (CGM) in an outpatient setting within 14 days before or after ICA with IVUS. Two patients were excluded because of insufficient CGM data, and a total of 71 patients with known or suspected CAD finally underwent iMap-IVUS and CGM examinations. Written informed consent was obtained from all subjects, and the protocol was approved for use by the Human Studies Subcommittee of Yokkaichi Hazu Medical Center (reference number 84).

### Coronary angiography and imaging procedures

Selective invasive coronary angiography was performed using standard techniques after right and left intracoronary administration of 2.5 to 5 mg isosorbide dinitrate. Quantitative analysis of invasive coronary angiograms (QCA: QAngio XA 7.1.14.0, Medis Medical Imaging Systems, Leiden, Netherlands) was performed.

### Imaging analyses

#### Gray-scale and iMap-IVUS

Gray-scale coronary IVUS and iMap-IVUS were performed in the lesions with mild to moderate stenosis (QCA-assessed percent diameter stenosis < 50%) in previously untreated vessels [8]. Severe calcified lesions covering greater than 180° were excluded from the IVUS assessment. After administration of 2.5 to 5 mg isosorbide dinitrate, an IVUS catheter (2.6 Fr, 40 MHz, Atlantis SR Pro2, Boston Scientific, Natick, MA, USA) was advanced into the target vessel, with the transducer positioned as distal as possible to the target lesion, and withdrawn using a motorized pullback device at a speed of 0.5 mm/s for 10 mm at each plaque [9]. The consoles used were iLab ultrasound systems (Boston Scientific). Images were recorded on DVD-R disks for subsequent off-line IVUS analysis. Two independent experienced investigators who were unaware of the patient profiles performed the IVUS analysis using validated planimetry software (echoPlaque4, INDEC Systems Inc., Santa Clara, CA, USA). In the gray-scale IVUS analysis, the cross-sectional area (CSA) was traced manually. The lumen-intimal border was traced, and the lumen CSA was calculated. The external elastic membrane (EEM)-adventitial border was traced, and the EEM CSA was determined. Plaque CSA was calculated as the EEM CSA minus the lumen CSA. These IVUS parameters were measured at the minimum lumen CSA sites and reference sites. The minimum lumen CSA site was determined as the location of the smallest amount of lumen in all analyzed cross-sections [6]. The remodeling index was defined as the ratio of EEM CSA at the measured lesion (minimum luminal site) to reference EEM CSA (average of the proximal and distal reference segments). Subsequently, EEM and lumen CSA were manually traced at 0.5-mm intervals for 10 mm length in each plaque. EEM, lumen and plaque (EEM CSA − lumen CSA) volumes were calculated using Simpson’s method. The percent plaque volume was calculated as (plaque volume/EEM volume) × 100 (%). To obtain iMap images, the integrated backscatter values for each tissue component were expressed in decibels and calculated using a fast-Fourier transform of the frequency component of the backscattered signal from a small volume of tissue. In the iMap IVUS analysis (Boston Scientific), plaque components were then classified as fibrotic (light-green), lipidic (yellow), necrotic (pink) or calcified (light-blue) [6]. Quantitative volumetric iMap IVUS analyses were performed to calculate fibrotic, lipidic, necrotic, and calcified volumes from the sum of fibrotic, lipidic, necrotic, and calcified areas in each CSA at 0.5-mm intervals for the iMap images. Percent plaque volumes were automatically calculated as each plaque component volume/plaque volume × 100 (%). In patients who underwent multi-vessel IVUS examination, the mean values of fibrotic, lipidic, necrotic and calcified plaque components of multiple plaques were calculated.

#### Biochemical markers

The blood samples were transported to the clinical laboratory of Yokkaichi Hazu Medical Center for further processing and storage at a temperature of − 80 °C within 0.5 h after blood collection. The eGFR of each patient was calculated from their serum creatinine (SCr) value and their age using the following equation: eGFR (mL/min/1.73 m^2^) = 194 × Age^− 0.287^ × SCr^− 1.094^ (if female × 0.739) [10]. Frozen EDTA-plasma samples were transported under controlled conditions (at − 80 °C) to Tokyo, Japan, where the concentrations of tumor necrosis factor (TNF)-α, interleukin-6, high-sensitivity C-reactive protein, adiponectin, malondialdehyde-modified low-density lipoprotein, total homocysteine, asymmetric dimethylarginine and 8-iso-prostaglandin F2α were determined using a validated multiplex assay (SRL, Tokyo, Japan). Proteinuria (−, ± , 1 + , or ≥ 2 +) was assessed using a dipstick test for spot-urine (Uropaper III; Eikenkagaku, Japan), and was defined as present if the dipstick result was ≥ 1 + [11].

#### CGM system measurement

CGM was performed for 7 consecutive days in all patients, and the daily glucose profiles were analyzed using data obtained from day 2 to day 6 to avoid any bias due to either insertion or removal of the sensor. The CGM analysis software (CareLink iPro, Medtronic, Northridge, California) calculated the measured variables: 120-h mean glucose levels, the time in hyperglycemia/hypoglycemia and the mean amplitude of glycemic excursions (MAGE) [12], which represents fluctuations in blood glucose levels over a 24-h period and was calculated from the daily variations in blood glucose level, measured continuously by CGM over a period of 5 days. Times in hyperglycemia and hypoglycemia were defined as the times when blood glucose levels were ≥ 200 and < 70 mg/dl, respectively [13]. All patients had daily meals during CGM.

### Statistical analysis

Categorical variables are presented as percentage frequencies and were analyzed using χ^2^ tests or Fisher exact tests as appropriate. Continuous normally distributed variables are expressed as mean ± SD and were compared using the Student’s two-tailed unpaired *t* test. Continuous data not normally distributed are expressed as median and interquartile range (IQR) and were analyzed using Mann–Whitney U tests. The Kolmogorov–Smirnov test was used to check for a normal distribution. Bivariate correlations between study variables were calculated using Spearman rank correlation coefficients. A one-way analysis of variance (ANOVA) with Tukey posthoc analysis was used for multiple comparisons. All statistical analyses were performed with SPSS version 19 (SPSS, Chicago, IL). Two-sided *p* values < 0.05 were considered to indicate statistical significance.

## Results

### Patient characteristics

Baseline patient characteristics on admission are shown in Table 1. The average age was 68 ± 9 years, and the majority of patients were men among the group of 71 study participants. We divided the patients into two groups according to their eGFR levels: Group 1 (≥ 60 mL/min/1.73 m^2^, n = 40) and Group 2 (< 60 mL/min/1.73 m^2^, n = 31). There were 3 patients with eGFR < 30 mL/min/1.73 m^2^ in Group 2. Group 2 patients were older and had a higher rate of hypertension than those in Group 1. Antihypertensive, antiplatelet and statin medications on admission did not differ between the two groups. Regarding diabetes medications, only pioglitazone and metformin were more frequently prescribed in Group 1 than in Group 2 (Additional file 1: Table S1). Fasting plasma glucose and HbA1c values were 121.2 ± 23.5 mg/dl and 7.4 ± 1.0% among all 71 study participants, and these values were not different between the two patient groups. Uric acid was higher in Group 2 than in Group 1. Group 2 had a higher rate of proteinuria than Group 1.

**Table 1:** Patient characteristics

Variables	Overall (n = 71)	Group 1 (n = 40)	Group 2 (n = 31)	*p* value
Age, years	68 ± 9	66 ± 9	71 ± 9	0.027
Male, n (%)	56 (79)	34 (83)	22 (71)	0.154
BMI, kg/m^2^	24.0 ± 3.6	24.3 ± 4.0	23.5 ± 2.9	0.343
Duration of DM, years	15.3 ± 8.8	14.4 ± 9.8	16.3 ± 7.4	0.350
Prior PCI, n (%)	38 (54)	23 (56)	15 (48)	0.448
Hypertension n (%)	60 (85)	29 (71)	31 (100)	0.002
Dyslipidemia, n (%)	49 (69)	26 (63)	23 (74)	0.409
Current smoker, n (%)	25 (35)	15 (37)	10 (32)	0.649
LVEF, %	65 ± 11	67 ± 8	63 ± 13	0.440
Laboratory data				
Serum creatinine, mg/dl	0.92 ± 0.29	0.76 ± 0.13	1.12 ± 0.32	< 0.001
eGFR, ml/min/1.73 m^2^	65.5 ± 19.1	78.3 ± 14.2	49.1 ± 10.0	< 0.001
Cholesterol, mg/dl				
Total	175.8 ± 31.9	177.8 ± 33.3	173.4 ± 30.4	0.556
HDL	47.4 ± 13.0	48.3 ± 13.1	46.4 ± 13.0	0.430
LDL	97.6 ± 26.2	100.3 ± 27.7	94.1 ± 24.1	0.326
Triglyceride, mg/dl	153.4 ± 89.9	155.1 ± 97.4	151 ± 80.7	0.724
Uric acid, mg/dl	5.9 ± 1.5	5.5 ± 1.5	6.4 ± 1.3	0.010
BNP, pg/ml	23.7 (9.1–72.3)	19.9 (10.2–42.9)	29.1 (9.1–83.0)	0.128
Conventional glucose indicators				
Fasting plasma glucose, mg/dl	121.2 ± 23.5	117.9 ± 21.3	125.4 ± 25.7	0.188
HbA1c, %	7.4 ± 1.0	7.3 ± 1.2	7.4 ± 0.8	0.180
1,5-AG, μg/ml	12.9 ± 8.4	12.0 ± 9.0	13.9 ± 7.6	0.226

Values are mean ± SD or number of patients (%). Group 1: ≥ 60 mL/min/1.73 m^2^, Group 2: < 60 mL/min/1.73 m^2^. *p* values for Group 1 vs. Group 2

*BMI* body mass index; *DM* diabetes mellitus; *PCI* percutaneous coronary intervention; *LVEF* left ventricular ejection fraction; *eGFR* estimated glomerular filtration rate; *HDL* high-density lipoprotein; *LDL* low-density lipoprotein; *BNP* brain natriuretic peptide; *HbA1c* hemoglobin A1_c_; *1,5-AG* 1,5-anhydroglucitol

### CGM parameters

Variables measured by the CGM system are shown in Table 2. Although maximum blood glucose was higher in Group 2 than in Group 1, other parameters including MAGE were similar between the two groups. During CGM, blood glucose values largely remained within the range of 90–200 mg/dl as recommended by the guideline [14]; however, various glucose variability patterns were observed and asymptomatic hyperglycemia and hypoglycemia were found in some patients in both groups to the same extent.

**Table 2:** Variables measured by the continuous glucose monitoring system

Variables	Overall (n = 71)	Group 1 (n = 40)	Group 2 (n = 31)	*p* value
Maximum blood glucose, mg/dl	274.6 ± 58.2	262.6 ± 49.9	290.2 ± 64.9	0.046
Minimum blood glucose, mg/dl	71.0 ± 24.7	71.1 ± 21.2	70.9 ± 29.0	0.566
Mean blood glucose, mg/dl	151.3 ± 35.8	145.0 ± 29.5	159.6 ± 41.6	0.154
MAGE, mg/dl	78.9 ± 24.9	75.2 ± 22.1	83.6 ± 27.7	0.242
Time in Hyperglycemia, %	9.6 (3.8–25.2)	8.8 (3.2–13.8)	12.7 (4.4–30.8)	0.344
Time in Hypoglycemia, %	0 (0–2.2)	0 (0–1.5)	0 (0–2.6)	0.591
Time in hyperglycemia, hours	10.6 (3.1–27.0)	8.8 (2.3–15.4)	15.2 (5.3–37.0)	0.178
Time in hypoglycemia, hours	0 (0–2.7)	0 (0–1.6)	0 (0–3.1)	0.599

Values are mean ± SD (%). Group 1: ≥ 60 mL/min/1.73 m^2^, Group 2: < 60 mL/min/1.73 m^2^. *p* values for Group 1 vs. Group 2

Time in hyperglycemia was defined as the time when blood glucose levels were equal to or greater than 200 mg/dl. Time in hypoglycemia was defined as the time when blood glucose levels were under 70 mg/dl. MAGE, mean amplitude of glycemic excursion

### Quantitative parameters of QCA, gray-scale and iMap-IVUS

A total of 153 plaques were assessed in 71 patients. Twenty-nine patients underwent a 3-vessel IVUS examination (15 patients in Group 1 and 14 patients in Group 2), whereas 24 patients underwent a 2-vessel IVUS examination (12 patients in Group 1 and 12 patients in Group 2) and 18 patients underwent 1-vessel IVUS examination (13 patients in Group 1 and 5 patients in Group 2). Table 3 shows quantitative QCA and IVUS findings. There were no significant differences in reference diameter, plaque location and percentage of diameter stenosis in each vessel between Group 1 and Group 2. In gray-scale IVUS, there were no significant differences in EEM CSA, lumen CSA, plaque CSA and remodeling index between the two groups. In addition, there were no significant differences in EEM volume, lumen volume, plaque volume and percent plaque volume between the two groups. Although absolute fibrotic volume was significantly higher in Group 1 than in Group 2, percent fibrotic volume (%FV), percent lipidic volume (%LV), percent necrotic volume (%NV) and percent calcified volume (%CV) were similar in the two groups.

**Table 3:** **IVUS**

Variables	Overall (n = 71)	Group 1 (n = 40)	Group 2 (n = 31)	*p* value
QCA analysis				
Reference diameter, mm	2.7 ± 0.7	2.6 ± 0.6	2.8 ± 0.8	0.186
Diameter stenosis, %	21.9 ± 12.9	23.6 ± 13.2	19.8 ± 12.2	0.078
Plaque location, n (%)				
LAD	51 (33.3)	27 (32.9)	24 (33.8)	0.360
LC	54 (35.3)	28 (34.2)	26 (36.6)	0.177
RCA	48 (31.4)	27 (32.9)	21 (29.6)	0.983
Diameter stenosis, %				
LAD	22.1 ± 12.5	23.6 ± 14.0	20.4 ± 10.6	0.361
LC	23.3 ± 16.8	24.8 ± 14.0	20.2 ± 13.1	0.219
RCA	21.1 ± 11.7	20.9 ± 11.1	21.3 ± 12.6	0.906
Gray-scale IVUS analysis				
EEM CSA, mm^2^	14.0 ± 4.9	14.0 ± 4.8	14.1 ± 5.0	0.953
Lumen CSA, mm^2^	7.8 ± 3.7	7.5 ± 3.4	8.2 ± 3.9	0.284
Plaque CSA, mm^2^	6.2 ± 2.5	6.5 ± 2.6	5.8 ± 2.4	0.109
Remodeling index	0.81 ± 0.22	0.83 ± 0.25	0.78 ± 0.20	0.181
EEM volume, mm^3^	140.3 ± 48.9	139.9 ± 48.1	140.7 ± 50.0	0.953
Lumen volume, mm^3^	78.4 ± 36.5	74.9 ± 34.3	82.4 ± 38.8	0.282
Plaque volume, mm^3^	61.9 ± 25.2	65.0 ± 25.7	58.3 ± 24.3	0.109
Plaque volume, %	45.0 ± 12.4	47.2 ± 11.6	42.41 ± 12.9	0.070
iMap IVUS analysis				
Fibrous volume, mm^3^ (%)	38.4 ± 14.1 (64.6 ± 12.8)	40.2 ± 13.2 (63.6 ± 13.4)	36.3 ± 14.8 (65.6 ± 11.9)	0.027 (0.106)
Lipid volume, mm^3^ (%)	7.1 ± 4.4 (10.9 ± 3.5)	7.5 ± 4.6 (10.8 ± 3.4)	6.7 ± 4.2 (10.9 ± 3.7)	0.281 (0.421)
Necrotic volume, mm^3^ (%)	15.4 ± 12.2 (22.3 ± 9.6)	16.7 ± 12.4 (23.1 ± 10.1)	13.8 ± 11.8 (21.4 ± 8.9)	0.102 (0.109)
Calcified volume, mm^3^ (%)	1.5 ± 1.7 (2.3 ± 1.9)	1.9 ± 2.0 (2.5 ± 2.0)	1.1 ± 1.1 (2.0 ± 1.7)	0.063 (0.251)

Values are mean ± SD (%). Group 1: ≥ 60 mL/min/1.73 m^2^, Group 2: < 60 mL/min/1.73 m^2^. *p* values for Group 1 vs. Group 2. EEM CSA, lumen CSA, and plaque CSA were measured at the minimum lumen CSA site in each plaque

*IVUS* intravascular ultrasound; *QCA* quantitative coronary angiography; *LAD* left anterior descending artery; *LC* left circumflex artery; *RCA* right coronary artery; *EEM* external elastic membrane; *CSA* cross-sectional area

### Intra- and inter-observer variabilities for IVUS analysis

Intra- or interobserver intra-class correlation coefficients (ICCs) for the EEM volume, lumen volume, and plaque volume were 0.929 and 0.989, 0.989 and 0.986, and 0.988 and 0.957, respectively. The intra- and interobserver ICCs for the %FV, %LV, %NV and %CV were 0.987 and 0.974, 0.980 and 0.957, 0.981 and 0.970, and 0.959 and 0.963, respectively.

### Biochemical markers

Biochemical markers are shown in Table 4. TNF-α, total homocysteine and 8-iso PGF2α were significantly greater in Group 2 than in Group 1.

**Table 4:** Biochemical markers

Variables		Overall (n = 71)	Group 1 (n = 40)	Group 2 (n = 31)	*p* value
TNF-α, pg/ml	1.2 (0.9–1.75)		1.1 (0.9–1.5)	1.6 (0.9–1.9)	0.041
IL-6, pg/ml	1.8 (1.2–2.75)		1.8 (1.175–2.725)	2 (1.3–2.6)	0.812
hs-CRP, ng/ml	432 (243–781.5)		430 (260–812.5)	448 (227–717.5)	0.469
Adiponectin, μg/ml	7 (5.6–11.35)		6.7 (5.35–10.85)	8.2 (6.25–12.05)	0.219
MDA-LDL, U/l	83 (64.5–102.5)		77.5 (62.75–95.25)	85 (68–107.5)	0.321
Total homocysteine, nmol/ml	10.5 (8.85–13)		99.9 (8.55–12.375)	11.2 (9.9–14.65)	0.022
ADMA, μmol/l	0.46 ± 0.07		0.45 ± 0.07	0.47 ± 0.07	0.169
8-iso PGF2α, pg/ml	17 (13.5–23.5)		16 (13–19.25)	19 (15.5–27)	0.027

Values are mean ± SD. Group 1: ≥ 60 mL/min/1.73 m^2^, Group 2: < 60 mL/min/1.73 m^2^. *p* values for Group 1 vs. Group 2

*TNF* tumor necrosis factor; *IL-6* interleukin-6; *hs-CRP* high sensitivity C-reactive protein; *MDA-LDA* malondialdehyde LDL; *ADMA* asymmetric dimethylarginine; *PGF2α* prostaglandin F2α; other abbreviations as in Table 1

### Relationships of coronary plaque composition to clinical and laboratory variables

**Fig. 1:**
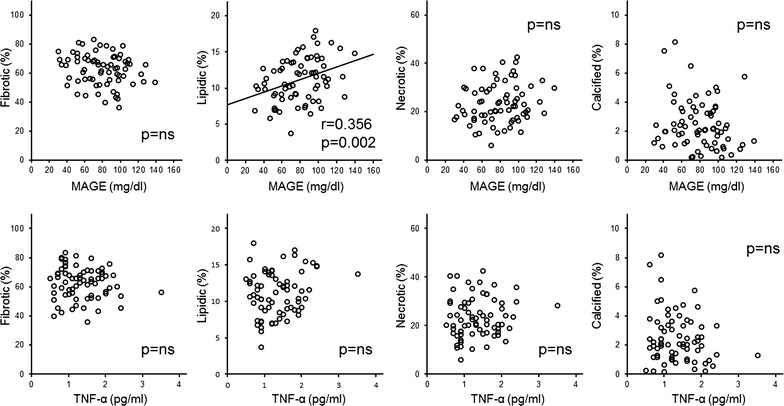
Correlation between mean amplitude of glycemic excursion (MAGE) and the percentage of tissue components (top) and tumor necrosis factor-α (bottom) in all patients. MAGE, mean amplitude of glycemic excursion; TNF-α, tumor necrosis factor-α; ns, not significant

**Fig. 2:**
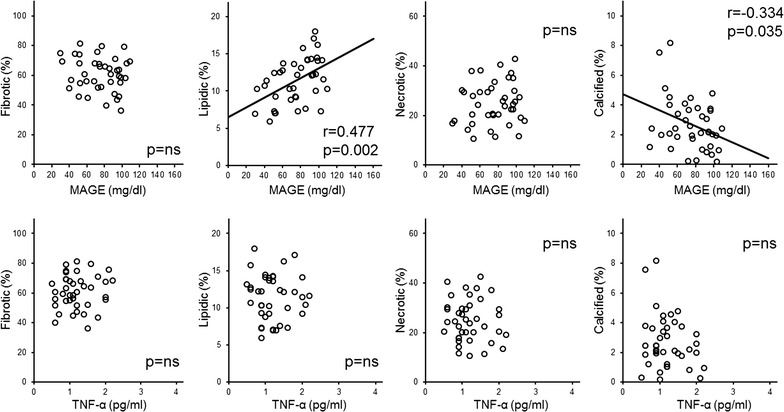
Correlation between mean amplitude of glycemic excursion (MAGE) and the percentage of tissue components (top) and tumor necrosis factor-α (bottom) in the Group 1. MAGE, mean amplitude of glycemic excursion; TNF-α, tumor necrosis factor-α; ns, not significant

**Fig. 3:**
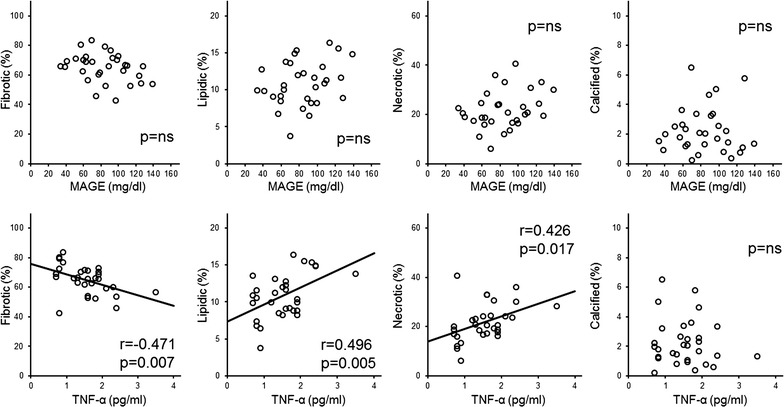
Correlation between mean amplitude of glycemic excursion (MAGE) and the percentage of tissue components (top) and tumor necrosis factor-α (bottom) in the Group 2. MAGE, mean amplitude of glycemic excursion; TNF-α, tumor necrosis factor-α; ns, not significant

For the whole patient population, there was a significant correlation only between %LV and MAGE (r = 0.356, p = 0.002), and stepwise multivariate linear regression analyses showed that only MAGE was independently associated with %LV (β = 0.356, p = 0.002, Table 5 and Fig. 1). In Group 1, %LV was positively correlated with MAGE (r = 0.477, p = 0.002) and HbA1c (r = 0.365, p = 0.020), and %CV was negatively correlated with MAGE (r = − 0.334, p = 0.035) (Table 6 and Fig. 2). Stepwise multivariate linear regression analyses showed that only MAGE was independently associated with %LV and %CV (%LV β = 0.477, p = 0.002, and %CV β = 0.334, p = 0.035) (Table 6). In contrast, %FV was negatively correlated with the serum level of TNF-α (r = − 0.471, p = 0.007), and %LV and %NV were positively correlated with the serum level of TNF-α (%LV: r = 0.496, p = 0.005, and %NV r = 0.426, p = 0.017) in Group 2 (Table 7 and Fig. 3). In this group, stepwise multivariate linear regression analyses showed that only the serum level of TNF-α was independently associated with %FV, %LV and %NV (%FV β = − 0.471, p = 0.007, %LV β = 0.496, p = 0.002, %NV β = 0.426, p = 0.017) (Table 7). However, eGFR had no significant correlation with serum TNF-α and coronary plaque characteristics in Group 2 (data not shown). The tissue characteristics of coronary plaque were not different between patients with and without proteinuria among all patients and within Groups 1 and 2 (data not shown).

**Table 5:** Univariate and multivariate linear regression for all patients

	Fibrotic				Lipidic				Necrotic				Calcified			
	Univariate		Multivariate		Univariate		Multivariate		Univariate		Multivariate		Univariate		Multivariate	
	Coefficients	p value	Coefficients	p value	Coefficients	p value	Coefficients	p value	Coefficients	p value	Coefficients	p value	Coefficients	p value	Coefficients	p value
Age	0.084	0.487	–	–	− 0.070	0.564			− 0.078	0.516	–	–	0.135	0.263	–	–
Duration of DM	− 0.039	0.747	–	–	0.049	0.688			0.048	0.690	–	–	0.030	0.806	–	–
Uric acid	− 0.049	0.686	–	–	0.102	0.399			0.056	0.642	–	–	− 0.142	0.236	–	–
HbA1c	− 0.050	0.681	–	–	0.177	0.140			0.069	0.566	–	–	− 0.146	0.223	–	–
Mean BG	− 0.173	0.149	–	–	0.206	0.085			0.160	0.181	–	–	0.043	0.725	–	–
MAGE	− 0.199	0.097	–	–	0.356	0.002	0.356	0.002	0.169	0.159	–	–	− 0.186	0.120	–	–
Time in hyperglycemia	− 0.204	0.089	–	–	0.334	0.004			0.183	0.127	–	–	− 0.099	0.413	–	–
Time in hypoglycemia	0.071	0.555	–	–	0.027	0.824			− 0.095	0.428	–	–	− 0.034	0.780	–	–
TNF-α	− 0.084	0.487	–	–	0.133	0.270			0.098	0.414	–	–	− 0.129	0.284	–	–
Total homocysteine	− 0.023	0.850	–	–	− 0.027	0.825			0.070	0.564	–	–	− 0.059	0.623	–	–
8-iso PGF2α	0.073	0.545	–	–	− 0.035	0.772			− 0.088	0.467	–	–	− 0.150	0.211	–	–

Group 1: ≥ 60 mL/min/1.73 m^2^, Group 2: < 60 mL/min/1.73 m^2^. Abbreviations are as shown in Tables 1, 2 and 4

**Table 6:** Univariate and multivariate linear regression for group 1

	Fibrotic				Lipidic				Necrotic				Calcified			
	Univariate		Multivariate		Univariate		Multivariate		Univariate		Multivariate		Univariate		Multivariate	
	Coefficients	p value	Coefficients	p value	Coefficients	p value	Coefficients	p value	Coefficients	p value	Coefficients	p value	Coefficients	p value	Coefficients	p value
Age	0.001	0.997	–	–	0.158	0.331	–	–	− 0.033	0.841	–	–	− 0.121	0.458	–	–
Duration of DM	− 0.027	0.869	–	–	0.096	0.556	–	–	0.002	0.992	–	–	0.000	0.998	–	–
Uric acid	− 0.098	0.549	–	–	− 0.014	0.933	–	–	0.139	0.392	–	–	− 0.010	0.949	–	–
HbA1c	− 0.156	0.337	–	–	0.365	0.020	–	–	0.145	0.372	–	–	− 0.319	0.045	–	–
Mean BG	− 0.056	0.733	–	–	0.212	0.188	–	–	− 0.004	0.981	–	–	0.010	0.951	–	–
MAGE	− 0.173	0.286	–	–	0.477	0.002	0.477	0.002	0.131	0.420	–	–	− 0.334	0.035	− 0.334	0.035
Time in hyperglycemia	− 0.084	0.605	–	–	− 0.290	0.069	–	–	0.025	0.879	–	–	− 0.077	0.638	–	–
Time in hypoglycemia	− 0.172	0.288	–	–	0.242	0.133	–	–	0.162	0.317	–	–	− 0.082	0.613	–	–
TNF-α	− 0.107	0.510	–	–	− 0.036	0.824	–	–	− 0.087	0.593	–	–	− 0.214	0.184	–	–
Total homocysteine	0.088	0.587	–	–	− 0.107	0.512	–	–	− 0.063	0.697	–	–	− 0.083	0.611	–	–
8-iso PGF2α	0.024	0.883	–	–	− 0.084	0.607	–	–	− 0.016	0.923	–	–	0.066	0.688	–	–

Group 1: ≥ 60 mL/min/1.73 m^2^, Group 2: < 60 mL/min/1.73 m^2^. Abbreviations are as shown in Tables 1, 2 and 4

**Table 7:** Univariate and multivariate linear regression for group 2

	Fibrotic					Lipidic				Necrotic				Calcified			
	Univariate		Multivariate			Univariate		Multivariate		Univariate		Multivariate		Univariate		Multivariate	
	Coefficients	p value	Coefficients	p value		Coefficients	p value	Coefficients	p value	Coefficients	p value	Coefficients	p value	Coefficients	p value	Coefficients	p value
Age	0.087	0.641	–		–	− 0.311	0.088	–	–	− 0.069	0.713	–	–	0.395	0.028	0.395	0.028
Duration of DM, years	− 0.088	0.636	–		–	− 0.097	0.602	–	–	0.103	0.581	–	–	0.232	0.208	–	–
Uric acid	− 0.146	0.434	–		–	0.382	0.034	–	–	0.093	0.618	–	–	− 0.286	0.119	–	–
HbA1c	− 0.210	0.258	–		–	0.128	0.493	–	–	0.202	0.275	–	–	0.065	0.728	–	–
Mean BG	− 0.307	0.093	–		–	0.217	0.241	–	–	0.288	0.117	–	–	0.079	0.673	–	–
MAGE	− 0.331	0.069	–		–	0.283	0.123	–	–	0.304	0.097	–	–	0.022	0.908	–	–
Time in hyperglycemia	− 0.316	0.083	–		–	0.220	0.235	–	–	0.293	0.110	–	–	0.107	0.566	–	–
Time in hypoglycemia	0.252	0.171	–		–	− 0.265	0.149	–	–	− 0.240	0.193	–	–	0.120	0.521	–	–
TNF-α	− 0.471	0.007	− 0.471		0.007	0.496	0.005	0.496	0.005	0.426	0.017	0.426	0.017	− 0.112	0.548	–	–
Total homocysteine	− 0.226	0.222	–		–	0.153	0.413	–	–	0.250	0.174	–	–	− 0.117	0.530	–	–
8-iso PGF2α	− 0.029	0.878	–		–	0.177	0.342	–	–	0.011	0.954	–	–	− 0.213	0.251	–	–

Group 1: ≥ 60 mL/min/1.73 m^2^, Group 2: < 60 mL/min/1.73 m^2^. Abbreviations as shown in Tables 1, 2 and 4

**Fig. 4:**
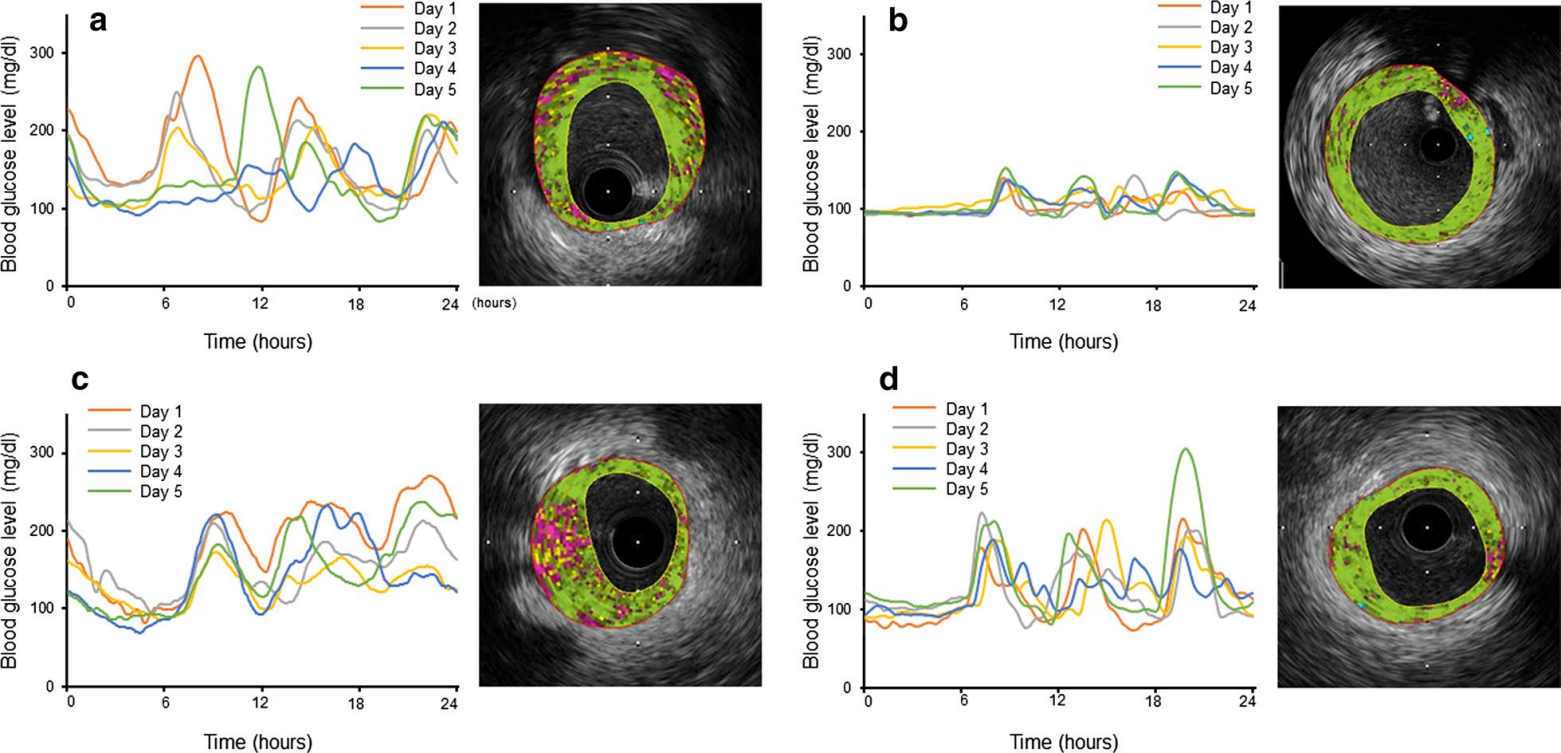
Representative daily variations in blood glucose level measured continuously by CGM over a period of 5 days (left) and iMap-coronary intravascular ultrasound images (right). **a** A patient with eGFR ≥ 60 ml/min/1.73 m^2^, high MAGE (98.5 mg/dl) and intermediate level of TNF-α (1.1 pg/ml). **b** A patient with eGFR ≥ 60 ml/min/1.73 m^2^, low MAGE (29.6 mg/dl) and intermediate level of TNF-α (1.2 pg/ml). **c** A patient with eGFR < 60 ml/min/1.73 m^2^, intermediate level of MAGE (74.8 mg/dl) and high TNF-α (2.4 pg/ml). **d** A patient with eGFR < 60 ml/min/1.73 m^2^, intermediate level of MAGE (84.3 mg/dl) and low TNF-α (0.8 pg/ml). The fibrotic, lipidic, necrotic and calcified components in each image were: **a** Fibrotic 58%, Lipidic 14%, Necrotic 26%, Calcified 2%. **b** Fibrotic 75%, Lipidic 7%, Necrotic 17%, Calcified 1%. **c** Fibrotic 46%, Lipidic 15%, Necrotic 36%, Calcified 3%. **d** Fibrotic 80%, Lipidic 7%, Necrotic 12%, Calcified 1%

Figure 4 shows representative CGM data over a period of 5 days (left) and iMap-coronary intravascular ultrasound images (right) in a patient with eGFR ≥ 60 ml/min/1.73 m^2^, high MAGE and intermediate level of serum TNF-α (A), a patient with eGFR ≥ 60 ml/min/1.73 m^2^, low MAGE and intermediate level of serum TNF-α (B), a patient with eGFR < 60 ml/min/1.73 m^2^, intermediate level of MAGE and high serum TNF-α (C) and a patient with eGFR < 60 ml/min/1.73 m^2^, intermediate level of MAGE and low serum TNF-α (D). Patient A had higher MAGE and greater lipidic and necrotic plaques than patient B. In contrast, patient C had similar MAGE but higher serum TNF-α and greater lipidic and necrotic plaques compared with patient D.

To investigate the synergistic effect of high MAGE and high TNFα on coronary plaque vulnerability, the 71 patients were divided into four groups according to the median values of MAGE (78.5 mg/dl) and TNF-α (1.2 pg/ml); Group A high MAGE and high TNF-α, n = 22, Group B high MAGE and low TNF-α, n = 14, Group C low MAGE and high TNF-α, n = 19, and Group D low MAGE and low TNF-α, n = 16. There was a statistically significant difference in %LV between groups as determined by one-way ANOVA (F(3,67) = 3.239, p = 0.027). A Tukey post hoc test revealed that Group A had greater %LV than Group D (Group A 12.2 ± 2.9%, and Group D 9.7 ± 2.8%, p < 0.05), but %LV was not different among Groups A, B (12.0 ± 3.3%) and C (10.6 ± 2.7%). In contrast, there were no statistically significant differences in %FV, %NV and %CV between groups.

## Discussion

The present study revealed that the factors that contribute to the tissue characteristics of coronary plaques differ depending on the renal function of patients with T2DM. As previously reported, the MAGE value strongly contributed to increased lipid plaque content among the whole patient population and within the patient group having eGFR ≥ 60 ml/min/1.73 m^2^ [3–5]. Interestingly, serum TNF-α but not MAGE strongly contributed to increased lipid and necrotic plaque contents and decreased fibrous plaque content in patients with reduced eGFR. Although all previous studies assessed the effects of MAGE on coronary plaque vulnerability by using CGM systems during hospitalization [1–5], our study demonstrated CGM in an outpatient setting over a period of 5 days to obtain information about real-life glycemic profiles.

T2DM is a well-known risk factor for CAD, and insulin resistance is strongly associated with the formation of high-risk lipid-rich vulnerable plaques [15, 16]. In addition, recent clinical studies have consistently shown that high glucose fluctuation strongly contributes to increased coronary plaque vulnerability both at the culprit lesion of ACS [1, 2] and unrelated lesions [3–5]. Kuroda et al. reported that glucose fluctuation expressed as MAGE played an important role in the progression of necrotic cores within the coronary plaque and formation of lipid-rich plaque with a thin-cap fibroatheroma in 70 patients with stable CAD pre-treated with lipid-lowering therapy [3]. It is recognized that inflammation and oxidative stress play pivotal roles in the progression of atherosclerosis and plaque instability [4, 17–19]. A recent experimental study in a rat model revealed that acute blood glucose fluctuation causes inflammation and oxidative stress in endothelial cells, increases the adhesion of monocytes to endothelial cells, and elevates endothelial cell apoptosis, resulting in cardiovascular injury [20]. Clinically, Rizzo et al. demonstrated that MAGE reduction achieved with dipeptidyl peptidase-IV inhibitors is associated with reduction of oxidative stress and markers of systemic inflammation in T2DM [21]. However, no clinical studies have demonstrated the synergistic effect of MAGE and inflammation and/or oxidative stress on coronary plaque vulnerability. Consistent with previous observations, the present study showed that MAGE values were significantly and positively correlated with lipid plaque content among the whole patient population and within the patient group with eGFR ≥ 60 ml/min/1.73 m^2^. However, there was no relationship between MAGE and plasma concentrations of inflammation and oxidative stress markers.

Chronic kidney disease (CKD) is another cause of systemic inflammation and oxidative stress and is recognized as an independent risk factor for CAD. Several investigators have studied the effects of renal function on coronary plaque characteristics [22–27]. Kawai et al. showed that the severity of coronary artery stenosis was higher in mild CKD patients with eGFR between 30 and 59 ml/min/1.73 m^2^, although there was no significant difference in the prevalence of high-risk plaque when compared to patients with eGFR ≥ 60 ml/min/1.73 m^2^ [22]. In contrast, other previous studies demonstrated that lower eGFR levels were associated with greater lipid and lower fibrous contents both in the target lesion of PCI [23] and non-target lesions with percent diameter stenosis < 50% [24]. Kato et al. also demonstrated that patients with CKD had a larger lipid index compared with non-CKD patients and that a lower eGFR was an independent risk factor for a larger lipid index [25]. The prevalence of T2DM in their studies was less than 50%, and T2DM can confound the relationship between the tissue characteristics of coronary plaque and renal function. The tissue characteristics of coronary plaque were not different between patients with preserved and reduced eGFR among the T2DM population in the present study. CKD also strongly contributes to coronary calcification, especially in patients with advanced CKD and end-stage renal disease [26, 27], but the effects of CKD on the tissue characteristics of coronary plaque can vary depending on whether the patient has T2DM and the stage of CKD. The degree of coronary calcification was similar between patients with eGFR ≥ 60 ml/min/1.73 m^2^ and those with eGFR < 60 ml/min/1.73 m^2^ in the present study, presumably because only a few patients had advanced CKD with eGFR < 30 ml/min/1.73 m^2^. Proteinuria is another important determinant of the risk of cardiovascular disease and mortality and may contribute to coronary plaque vulnerability. Shimbo et al. demonstrated that patients with proteinuria had coronary plaques with significantly greater percentage lipid volume compared with those without, and that the presence of proteinuria is an independent predictor for lipid-rich plaque [11]. About 44% of patients had T2DM in their studies, and the rate of T2DM was much higher in patients with proteinuria than among those without it. Although T2DM itself was not identified as an independent predictor for lipid-rich plaque, T2DM can confound the relationship between tissue characteristics of coronary plaque and proteinuria. The tissue characteristics of coronary plaque were not different between patients with and without proteinuria among the whole patient population and even within the two eGFR groups in the present study, presumably because all study participants had T2DM. In addition, severe calcified lesions covering greater than 180° were excluded from the IVUS assessment, which can influence the relationship between coronary calcification and CKD.

No previous clinical studies have taken into account eGFR in the relationship between MAGE and coronary plaque characteristics. The present study revealed for the first time that the MAGE value strongly contributes to increased lipid plaque content only in patients with eGFR ≥ 60 ml/min/1.73 m^2^, and we found that patients with eGFR < 60 ml/min/1.73 m^2^ had significantly higher serum levels of TNF-α than those with eGFR ≥ 60 ml/min/1.73 m^2^. Interestingly, the serum level of TNF-α but not MAGE was identified as an independent contributor to increased lipid and necrotic plaque contents and decreased fibrous plaque content in patients with eGFR < 60 ml/min/1.73 m^2^. These results may indicate that the influence of glucose fluctuation on coronary plaque formation is attenuated in the setting of CKD, or that CKD-related inflammation surpasses the MAGE-related inflammatory process in the progression of coronary plaque formation. TNF-α is a well-known potent pro-inflammatory cytokine involved in the pathogenesis of arteriosclerosis, and plays a pivotal role in orchestrating the cytokine cascade in many inflammatory diseases because of this role as a “master-regulator” of inflammatory cytokine production [28]. Kim et al. induced atherosclerotic plaque in 24 iliac arteries from 12 rabbits with a combination of a high cholesterol diet, endothelial denudation, and injection into the vessel wall of either saline (n = 5), olive oil (n = 6), or inflammatory proteins [n = 13, high-mobility group protein B1 (HMGB1) n = 8 and TNF-α n = 5]. They found that macrophage infiltration was present to a higher degree in the HMGB1 and TNF-α groups, compared with the oil or saline groups. In addition, lipid rich plaques were more frequently detected in the inflammatory protein group on optical coherence tomography [29]. They concluded that advanced atheromatous plaques, including lipid rich plaques, were more frequently induced by the injection of pro-inflammatory proteins, compared to the controls. Furthermore, the pro-inflammatory groups showed increased expression of TNF-α and HMGB1 within the intima and media of the vessel, compared with either the saline or oil injected groups. These findings suggest that direct tissue injection of pro-inflammatory proteins may induce potent inflammation and promote the development of an advanced atheromatous plaque. CKD is one of the most important factors associated with a significant increase in TNF-α activity [30] and influences macrophage behavior [31]. Interestingly, a very recent study demonstrated that the plasma levels of circulating TNF receptors (cTNFRs) are significantly increased in CKD patients and are closely correlated with kidney function [32]. Because eGFR had no significant correlation with TNF-α and coronary plaque characteristics in patients with < 60 ml/min/1.73 m^2^, the increased expression of circulating and/or coronary plaque TNFRs may explain the strong association between plasma TNF-α and tissue characteristics of coronary plaques only in patients with reduced eGFR. Although the results in the present study indicated no synergistic effect of high MAGE and high TNFα on coronary plaque vulnerability, further study is warranted.

## Study limitations

In terms of the limitations of this study, first, this single-center study enrolled only a small number of subjects, which limited clinical interpretation. Second, the time course of progression of coronary atheromatous plaques was not examined in the present study. Third, expressions of inflammatory cytokines and their receptors in the coronary plaques were not assessed. Finally, the duration of CKD was not assessed although it can closely link to chronic inflammatory processes and may contribute to the progression of coronary atheromatous plaques.

## Conclusions

The factors that contribute to tissue characteristics of coronary plaques differ depending on the renal function of patients with T2DM. MAGE in patients with eGFR ≥ 60 mL/min/1.73 m^2^ and serum TNF-α in those with eGFR < 60 mL/min/1.73 m^2^ were independently associated with coronary plaque vulnerability.

## Additional file

**Additional file 1: Table S1.** Medications.

## Abbreviations

T2DM: type 2 diabetes mellitus
ACS: acute coronary syndrome
IVUS: intravascular ultrasound
ICA: invasive coronary angiography
CAD: coronary artery disease
CGM: continuous glucose monitoring
eGFR: estimated glomerular filtration rate
CSA: cross-sectional area
EEM: external elastic membrane
TNF: tumor necrosis factor
MAGE: mean amplitude of glycemic excursions
IQR: interquartile range
ICCs: intra-class correlation coefficients
%LV: percent lipidic volume
%CV: percent calcified volume
%FV: percent fibrotic volume
%NV: percent necrotic volume
CKD: chronic kidney disease
HMGB1: high-mobility group protein B1
cTNFRs: circulating tumor necrosis factor receptors
