# Supplementary material for: Impact of renal function on the underlying pathophysiology of coronary plaque composition in patients with type 2 diabetes mellitus
Source: Cardiovasc Diabetol. 2017 Oct 12;16:131. doi: 10.1186/s12933-017-0618-3 (PMC5639771; doi:10.1186/s12933-017-0618-3)
Supplement: Supplementary file 1 — Additional file 1: Table S1. Medications. [file 12933_2017_618_MOESM1_ESM.docx]

**Supplemental Table 1: Medications**

|  | Overall (n =71) | Group 1 (n =40) | Group 2 (n =31) | *p*-value |
| --- | --- | --- | --- | --- |
| ACE-I/ARB, n (%) | 59 (83) | 31 (76) | 28 (90) | 0.156 |
| CCB, n (%) | 26 (37) | 13 (32) | 13 (42) | 0.416 |
| β-blocker, n (%) | 12 (17) | 5 (12) | 7 (23) | 0.264 |
| Statin, n (%) | 45 (63) | 26 (65) | 19 (61) | 0.749 |
| Ezetimibe, n (%) | 5 (7) | 1 (2) | 4 (13) | 0.098 |
| Aspirin, n (%) | 42 (59) | 25 (61) | 17 (55) | 0.518 |
| Thienopyridine, n (%) | 17 (24) | 10 (24) | 7 (23) | 0.814 |
| SU, n (%) | 24 (34) | 15 (37) | 9 (30) | 0.458 |
| α-GI, n (%) | 15 (21) | 7 (18) | 8 (27) | 0.398 |
| Pioglitazone, n (%) | 6 (8) | 6 (15) | 0 (0) | 0.025 |
| Metformin, n (%) | 20 (28) | 15 (37) | 5 (16) | 0.049 |
| DPP4-I, n (%) | 44 (62) | 25 (61) | 19 (61) | 0.918 |
| Insulin, n (%) | 13 (18) | 5 (12) | 8 (26) | 0.153 |

Group 1: ≥60 mL/min/1.73m², Group 2: <60 mL/min/1.73m². *p*-values for Group 1 vs. Group 2.

ACE-I, angiotensin-converting enzyme inhibitor; ARB, angiotensin II receptor blocker; CCB, calcium channel blocker; SU, sulfonylurea; α-GI, α-glucosidase inhibitor; DPP4-I, dipeptidyl peptidase-4 inhibitor.
